# New Insights to Adenovirus-Directed Innate Immunity in Respiratory Epithelial Cells

**DOI:** 10.3390/microorganisms7080216

**Published:** 2019-07-25

**Authors:** Cathleen R. Carlin

**Affiliations:** Department of Molecular Biology and Microbiology and the Case Comprehensive Cancer Center, School of Medicine, Case Western Reserve University, Cleveland, OH 44106, USA; cathleen.carlin@case.edu; Tel.: +1-216-368-8939

**Keywords:** adenovirus, early region 3, innate immunity, NFκB

## Abstract

The nuclear factor kappa-light-chain-enhancer of activated B cells (NFκB) family of transcription factors is a key component of the host innate immune response to infectious adenoviruses and adenovirus vectors. In this review, we will discuss a regulatory adenoviral protein encoded by early region 3 (E3) called E3-RIDα, which targets NFκB through subversion of novel host cell pathways. E3-RIDα down-regulates an EGF receptor signaling pathway, which overrides NFκB negative feedback control in the nucleus, and is induced by cell stress associated with viral infection and exposure to the pro-inflammatory cytokine TNF-α. E3-RIDα also modulates NFκB signaling downstream of the lipopolysaccharide receptor, Toll-like receptor 4, through formation of membrane contact sites controlling cholesterol levels in endosomes. These innate immune evasion tactics have yielded unique perspectives regarding the potential physiological functions of host cell pathways with important roles in infectious disease.

## 1. Introduction

Adenoviruses have proven to be invaluable experimental tools contributing to many breakthrough discoveries, including mRNA splicing and antigen presentation to T cells [1,2]. The finding that adenovirus type 12 caused cancer in hamsters in a laboratory setting was the first example of oncogenic activity by a human virus [3]. Although adenoviruses have not been linked to human tumors, they have led to many advances in cancer biology, particularly in the field of tumor suppressors [4]. The study of host cellular pathways required for the adenovirus life cycle, and host restriction factors limiting its replication, has led to many significant advances in eukaryotic cell and molecular biology [5,6,7,8]. Adenovirus early region 3 (E3) gene products in particular have been powerful tools for discovering new mechanisms in the field of intracellular membrane trafficking [9,10,11]. Interactions between adenovirus E3 proteins and components of the host immune system have also revealed important insights into immune evasion tactics that are widely employed by intracellular pathogens [12,13]. In this review, we describe several new host cell pathways linking E3 to pro-inflammatory NFκB signaling in respiratory epithelial cells.

## 2. Human Adenoviruses

Human adenoviruses were first isolated from adenoid tissues removed from healthy children in 1953 [14]. A total of 90 genotypes divided into seven subgroups (A–G) have now been identified, including the original 51 serotypes and emergent recombinant species associated with recent outbreaks of adenovirus disease [15,16,17]. Adenovirus infections commonly present with mild respiratory or gastrointestinal symptoms in children under the age of five [18]. Other clinical manifestations include keratoconjunctivitis, hepatitis, and myocarditis [19]. Although most infections are self-limiting, periodic adenovirus outbreaks can pose significant health risks in people with no known predisposing conditions [20,21,22]. For instance, acute respiratory disease (ARD) is a serious and frequently fatal condition caused by adenovirus infections during conditions of crowding and stress [12,13,23]. Adenoviruses are also emerging as significant pathogens associated with high morbidity and mortality in individuals with weakened immune systems such as transplant patients, and people with existing respiratory or cardiac disease [18,24,25,26,27,28]. Currently, there are no FDA-approved drugs that are adenovirus-specific [29]. Although the antiviral drug cidofovir is used in transplant patients with probable adenovirus disease, the potential benefit of cidofovir therapy must be weighed against a relatively high risk of nephrotoxicity [30,31]. Brincidofovir is a lipid conjugate of cidofovir with increased antiviral potency against adenovirus and other double-stranded DNA viruses, which does not appear to be nephrotoxic [32]. Although brincidofovir is not yet FDA-approved, the drug is available for clinical use in transplant patients with suspected adenovirus disease through an expanded use protocol (https://www.clinicaltrials.gov/ct2/show/NCT02596997). A vaccine for adenovirus types 4 and 7 has been licensed for use by the US military because of high potential for ARD [33]. Recent public health concerns have raised the issue of whether the adenovirus vaccine should also be made available to the general public [34].

Adenoviruses are non-enveloped icosahedral viruses with a non-segmented double-stranded (ds) DNA genome that is approximately 36-kb in size [23,35,36]. The vertices of the icosahedral adenovirus capsid are comprised of a pentameric penton base and trimeric fiber proteins that mediate viral attachment and internalization [37]. Adenoviruses encode approximately 40 genes, which are read from both strands of the viral DNA [23,35,36]. The adenovirus life cycle is separated into an early and a late phase by the DNA replication process [23]. The early transcription units (E1, E2, E3, E4) mainly encode non-structural, regulatory proteins that are necessary for DNA synthesis, activate other virus genes, and prevent premature death of the infected cell by host innate and adaptive immunity defenses [36]. E3 transcripts produce several proteins with the capacity to promote persistent infections by modulating host immune responses [9]. Although they have been studied primarily in group C adenoviruses (HAdV-C2 and HAdV-C5), these immunomodulatory proteins are conserved across all (E3-RIDα or E3-10.4K, E3-RIDβ or E3-14.5K, and E3-14.7K), or a great majority (E3-19K) of adenovirus species [38]. Other E3 products, such as the secreted 49K protein encoded by group D adenoviruses, are uniquely expressed [39]. The late phase of the adenovirus life cycle is focused on production of structural proteins to package newly replicated viral DNA [23]. Once assembled in the nucleus, viral progeny are released by cell lysis through autophagy and autophagy-triggered caspase activity [40].

Adenovirus vectors have been extensively developed for gene therapy, as vaccines, and for cancer therapy over the past few decades, accounting for approximately a quarter of gene therapy clinical trials world-wide [41,42]. However, adenovirus vectors activate the innate immune system, representing a major barrier to the effective use of these agents in gene correction applications [12,13]. On the other hand, the innate immune response to adenovirus is a potentially useful attribute for vaccine development and cancer immune therapy [12]. This has motivated intensive efforts to understand the molecular basis of adenovirus-induced innate immunity, and how the virus strikes a balance between the elimination of virus and immune-mediated tissue injury [43]. These opposing regulatory mechanisms are primarily mediated by viral cell entry, early adenovirus proteins, and two virus-associated non-coding RNAs VA-I and VA-II [13]. Many of these mechanisms converge on the nuclear factor kappa-light-chain-enhancer of activated B cells (NFκB) pathway [12,13,44].

## 3. Adenoviral Cell Entry and Innate Immunity

**Figure 1:**
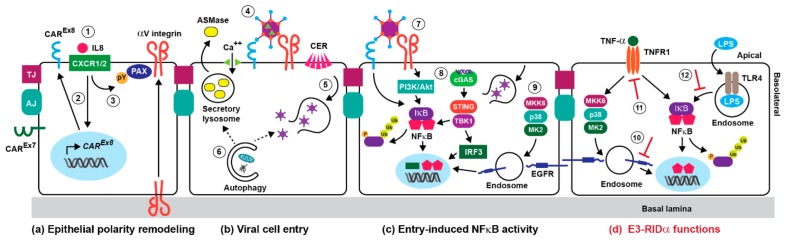
Adenovirus-directed innate immunity through nuclear factor kappa-light-chain-enhancer of activated B cells (NFκB). (**a**) Adenovirus induces host cell secretion of IL-8 (1), which regulates epithelial polarity of Car^Ex8^ (2) and αV integrins (3), allowing adenovirus entry from apical surface of respiratory epithelial cells. (**b**) Host cell receptor binding also unmasks the viral membrane lytic protein-VI (green triangle), which activates a membrane repair system leading to the generation of plasma membrane ceramide lipids (4). Ceramides promote virus internalization and endosomal membrane penetration (5). Viral cell entry is also enhanced by elevated levels of autophagy via hypothetical mechanisms discussed in the text (6). (**c**) Adenovirus binding to host cell receptors elicits a first wave of NFκB activity (7). Endosomal penetration also contributes to NFκB activity through a cGAS-STING cytosolic DNA sensing pathway (8), and a p38-MAPK signaling cascade capable of activating EGFR (EGF receptor) stress responses (9). (**d**) In this review we discuss evidence suggesting that E3-RIDα regulates NFkB activity occurring downstream of TNF-α, by antagonizing stress-induced EGFR signaling in endosomes (10), and promoting post-internalization TNFR1 sorting to lysosomes (11); and downstream of LPS, by limiting cholesterol-dependent TLR4 signaling from endosomes (12).

Viral cell entry requires interactions between the virus and two sets of host cell receptors. The first interaction is mediated by the knob domain of fiber proteins binding to the coxsackievirus adenovirus receptor (CAR) for all serotypes except those belonging to group B, which bind the CD46 complement regulatory protein [45,46]. Some serotypes also utilize sialic acid-containing glycoproteins, as primary receptors in addition to CAR [47,48]. This is followed by a secondary interaction between a motif in the penton base protein and the RGD (Arginine, Glycine, and Aspartate) domain of αV integrins [49]. Adenoviruses invade the lumen of the digestive and respiratory tracts. Paradoxically, adenovirus receptors are normally located on basolateral membranes of epithelial cells lining those tissues, representing a significant barrier to infection from the apical surface facing the lumen [50]. However, the initial innate immune response in alveolar macrophages appears to induce two pathways that regulate the epithelial polarity of these host cell receptors. The first pathway involves production of an alternatively spliced form of CAR (CAR^Ex8^) that is specifically targeted to the apical surface, as opposed to CAR^Ex7^ found on basolateral membranes (Figure 1a) [51]. This process is regulated by exposure to the proinflammatory cytokine and neutrophil chemoattractant IL-8, which binds CXCR1 and CXCR2 receptors located on apical epithelial surfaces. IL-8 binding induces an Akt/S6K (Serine/threonine-protein kinase) intracellular signaling pathway in epithelial cells, culminating in de novo protein synthesis and apical membrane targeting of CAR^Ex8^ [52]. Adenovirus entry into the epithelium appears to be further enhanced by neutrophils recruited to the apical surface through their interactions with CAR^Ex8^ [52]. In the second pathway, IL-8 activates a Src kinase-dependent mechanism causing apical membrane recruitment of the focal adhesion scaffolding protein paxillin (Figure 1a) [53]. Paxillin then appears to either function in apical trafficking of αvβ3 integrin, or as a component of a mechanism for retaining αvβ3 on the apical membrane [53]. Epithelial remodeling of αvβ3 polarity subsequently enables apical binding and infection with adenovirus following binding to the primary adenovirus receptor CAR^Ex8^ [53].

Internalized virions have been shown to co-localize with some endocytic markers, chiefly for early endosomes [54,55]. It was also known that adenovirus uncoating and release of the viral membrane lytic protein-VI were necessary to penetrate cell membranes, allowing the virus access to the cytoplasm [56]. However, the identity of the compartment mediating escape to the cytosol was elusive, until recent studies identifying a role for an evolutionarily conserved physiological process for repairing membrane damage [57]. These studies revealed that adenovirus undergoes partial uncoating exposing protein-VI, upon mechanical cues from CAR and αvβ3 integrin viral receptors [58,59]. Protein-VI subsequently induces small plasma membrane lesions, stimulating calcium influx and plasma membrane fusion of secretory lysosomes, which both repairs membrane damage and releases acid sphingomyelinase (ASMase) into the extracellular space (Figure 1b) [57,58]. ASMase then converts plasma membrane sphingomyelin into ceramide lipids, which enhances virus endocytosis and protein-VI-mediated membrane rupture (Figure 1b) [58]. Studies have also revealed that early adenovirus gene expression and virus production were both enhanced in respiratory epithelial cells with elevated autophagy, which is an important adaptive response to high oxygen pressure in the airway [60,61]. Although the role of autophagy in the early stages of infection is not currently known, it could maintain the homeostasis of the endosome/lysosome pathway by repairing endosomes that have been damaged by protein VI-mediated membrane rupture (Figure 1b) [62]. Alternatively, autophagy could participate in the plasma membrane repair process driving endocytic uptake of the virus [63]. Serotype 7 belonging to adenovirus group B escapes from late endosomes/lysosomes, suggesting that the primary viral receptor CD46 initiates viral uptake through a different mechanism [64].

**Figure 2:**
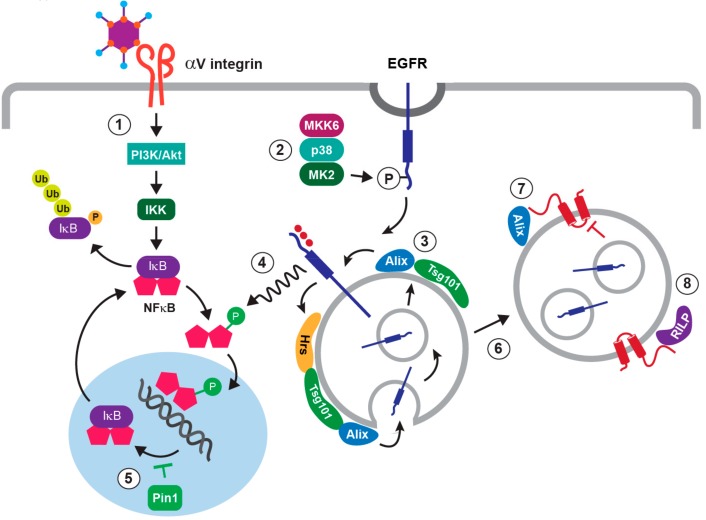
Hypothetical model for adenoviral regulation of stress-induced EGFR signaling through NFκB. We hypothesize that viral cell entry activates two host cell pathways converging on NFκB. Capsid binding to αV integrins induces PI3K/Akt, which activates a canonical NFκB signaling pathway by promoting degradation of inhibitory IκB proteins and also unmasks a Pin1 substrate in NFκB-p65 (1); and viral cell entry elicits clathrin-mediated EGFR uptake to a stable subset of non-degradative MVBs (multivesicular body endosomes) downstream of p38-MAPK signaling (2). Similar to other cellular stresses, we hypothesize that EGFRs cycle between ILVs and limiting membranes under regulation of ESCRT proteins Hrs, Tsg101, and Alix (3). We have shown that stress-activated EGFRs (EGF receptors) (red highlights) facilitate phosphorylation of the Pin1 NFκB-p65 substrate prior to its nuclear translocation (4), which is known to subsequently block nuclear export of IκB/NFκB complexes (5). E3-RIDα is produced 3 to 4 hours post-infection (6). Our published data support a working model that E3-RIDα antagonizes EGFR stress signaling from MVB limiting membranes (7); and promotes lysosomal degradation (8), by mechanisms involving host cell proteins Alix and Rab-interacting lysosomal protein (RILP).

As with other viruses such as HSV-1, NFκB activation appears to occur in waves that presumably induce different patterns of gene expression over the course of an acute adenovirus infection [65]. Capsid binding to host cell receptors represents the earliest mechanism for activating NFκB signaling during an acute infection (Figure 1c). For instance, the interaction between the adenovirus fiber and CAR has been reported to elicit intracellular signaling leading to cytokine expression in cultured human respiratory epithelial cells [66]. It has also been demonstrated that adenovirus vectors activate a phosphoinositide 3-kinase (PI3K)/Akt signaling pathway contributing to NFκB-dependent cytokine expression in cultured epithelial cells, following binding to αV integrins [67]. Once the virus has been internalized, endosomal escape activates another set of signaling pathways regulating host cell gene transcription before the onset of viral gene expression. Cytosolic adenovirus DNA is recognized by cyclic GMP-AMP synthase (cGAS), a DNA sensor that produces cyclic guanine-adenine dinucleotide (cGAMP) (Figure 1c) [68]. cGAMP then induces STING (Stimulator of interferon genes), a signaling molecule associated with the endoplasmic reticulum (ER) that activates IRF3 and NFκB transcription factors through an interaction with the IKK (inhibitor of NFκB kinases)-like kinase TBK1 (Figure 1c) [69]. Incoming adenovirus particles also activate p38-MAPK and its downstream effector MAPKAP kinase 2 (MK2), by a mechanism that is dependent on the p38-MAPK kinase MKK6, but independent of integrin-mediated cell signaling (Figure 1c) [70]. Capsid-induced p38-MAPK signaling could be activated as a response to the plasma membrane repair pathway mediating viral cell entry [71]. In addition to enhancing microtubule-mediated targeting of virus to the nuclear pore, p38-MAPK signaling has been linked to early inflammatory responses provoked by virus penetration of the endosome [70,72]. However, exactly how the p38-MAPK signaling cascade regulates NFκB signaling during an adenovirus infection was unknown. Our recent studies have suggested the involvement of a stress-activated EGFR (EGF receptor) signaling pathway, resulting in the stabilization and enhanced activity of nuclear NFκB in respiratory epithelial cells in vitro (Figure 2) [73].

## 4. The NFκB Pathway in Adenovirus-Directed Innate Immunity

NFκB is a key component of the initial innate immune with important roles in severe manifestations of acute adenovirus infections and toxicity elicited by adenovirus gene therapy vectors [12,13]. For instance, several lines of evidence suggest that early tissue injury associated with infectious adenoviruses and adenovirus vectors is largely due to NFκB-mediated neutrophil recruitment. It has been known for nearly three decades that infectious adenoviruses can specifically induce host cell secretion of chemokine IL-8, which attracts neutrophils to sites of infection, in animal models [43,74]. More recently, studies have shown that proinflammatory cytokines including IL-8 were strongly induced in children with symptomatic human adenovirus viremia after stem cell transplantation [75]. Adenovirus has also been investigated in the pathogenesis of COPD (chronic obstructive disease), where E1A expression has been potentially linked to increased NFκB activity and IL-8 expression in response to inflammatory stimuli [76]. In addition, intravenously administered adenoviral vectors significantly increased chemokine production, which correlated with acute neutrophil-mediated hepatic injury, in human adenovirus vector-exposed mice [77].

The NFκB family is comprised of multiple subunits (RelA/p65, c-Rel, RelB, p50 and p52), which assemble combinatorially into functioning homo- and heterodimers capable of dimer-specific gene activation [78]. NFκB subunits are sequestered in the cytoplasm as inactive dimers bound to inhibitory IκB proteins in resting cells (Figure 2) [79]. Most upstream stimuli activate NFκB by inducing phosphorylation-dependent proteasomal degradation of IκB proteins [79]. This activation step is primarily regulated by inducible IKKs, which are composed of a heterodimer of the catalytic IKKα and IKKβ subunits, and a regulatory protein called NEMO (NFκB essential modulator) or IKKγ [80]. Two IKK-related kinases have also been identified (TBK1 and IKKε). Primarily known for activating the transcription factors IRF3 and IRF7, which are critical for expression of type I interferon genes, TBK1 and IKKε are both reported to be NFκB-activating kinases [81]. The serine/threonine-specific protein kinase Akt also regulates NFκB by binding and activating IKKα [82]. Liberated NFκB dimers then translocate to the nucleus, where they bind specific DNA sequences via a conserved Rel homology domain (RHD) at their N-terminus.

One important mechanism for terminating NFκB responses involves de novo synthesized IκB proteins, which enter the nucleus, remove NFκB from DNA, and relocalize it to the cytosol where it is subsequently targeted for proteasomal degradation (Figure 2) [83]. It is also known that nuclear NFκB function is regulated by Pin1, a peptidyl-prolyl isomerase which controls protein function by catalyzing conformational changes at specific pSer/Thr-Pro motifs, which are substrates for proline-directed kinases such as ERK-MAPK [84]. Studies have revealed that cytokine treatment induces phosphorylation at the Thr254-Pro motif in the NFκB p65 subunit, leading to Pin1 binding following nuclear translocation, prolyl isomerization, inhibition of p65 binding to IκBα, and increased nuclear stability and activity of NFκB [85,86]. Deregulated Pin1 function has been implicated in various diseases, and Pin1 is being investigated as a potential therapeutic target in cancer and immune disorders [87,88,89]. Pin1 is also emerging as an important factor in viral pathogenesis. For instance, Pin1 has been reported to limit RNA virus–dependent tissue damage caused by type I interferons, by inducing proteasomal destruction of IRF3 transcription factor [90]. Recent studies have also revealed that Pin1 regulates stability and function of several virally-encoded phosphoproteins, including hepatitis B virus X protein, HTLV-1 Tax protein, and HIV integrase [91].

The NFκB signaling pathway promotes inflammation and immunity against pathogens by regulating the expression of an array of pro-inflammatory mediators, including cytokines and chemokines [79]. The NFκB-driven innate immune response to infectious adenovirus has been examined extensively in macrophages and epithelial cells, which are the first two cell types to engage adenovirus during an acute respiratory tract infection. Although alveolar macrophages are generally non-permissive for viral replication, several lines of evidence suggest that they elicit innate immune responses following adenovirus internalization. For instance, blood macrophages have been shown to produce inflammatory cytokines following exposure to adenovirus in vitro; and internalization of adenovirus by alveolar macrophages initiates early proinflammatory signaling during acute respiratory tract infection in vivo [92,93,94]. It has also been shown that the infectious adenovirus life cycle activates inflammatory signaling pathways in epithelial cells both in vitro and in vivo [44]. In vitro co-culture studies have revealed that the innate immune response to adenovirus is significantly enhanced through a synergistic interaction between macrophages and epithelial cells, by a mechanism requiring NFκB activity, as well as direct physical contact between these two cell types [95]. There is also evidence that basal and induced NFκB activity protects epithelial cells from the inflammation they induce by activating local innate immune responses [65].

## 5. Stress-Induced EGFR Responses

EGFR is activated by ligand binding at the cell surface, resulting in autophosphorylation on specific tyrosine residues within the cytoplasmic tail that serve as docking sites for a range of proteins regulating multiple intracellular signaling pathways [96]. Ligand-activated EGFRs are rapidly internalized to endosomes that enable signaling from unique intracellular platforms [97,98]. Endocytosis also instigates the process of signal termination since receptors are targeted for degradation by the ESCRT (endosomal sorting complexes required for transport) machinery, which recognizes ubiquitin moieties attached to EGFR at plasma membrane [99,100]. Briefly, ESCRT-0 comprised of Hrs and Stam1 subunits sequesters cargo in peripheral early endosomes, and ESCRT-I and ESCRT-II act sequentially to sort cargo into inward invaginations of limiting membranes of MVB (multivesicular body) endosomes [101]. ESCRT-III then facilitates cargo deubiquitination and membrane scission finalizing formation of ILVs (intraluminal vesicles), which are subsequently hydrolyzed in lysosomes following late endosome/MVB-lysosome fusion [102].

Recent studies have shown that a variety of cellular stresses, or stress inducers such as TNF-α, stimulate a robust, non-canonical pathway of ligand-independent EGFR trafficking, downstream of a p38-MAPK/MK2 signaling cascade (Figure 2) [103,104,105]. In contrast to ligand-stimulated EGFRs that are targeted for degradation, stress-internalized EGFRs arrest in a relatively stable population of MVB endosomes, where they are subsequently activated in the absence of ligand [103,104]. The stress-internalized EGFRs localize to ILVs capable of undergoing dynamic cycles of fission and fusion at the MVB limiting membrane, which is thought to facilitate EGFR stress signaling via cytosolic substrates [106]; and EGFR recycling back to plasma membrane when p38-MAPK activity declines [107]. Stress-induced EGFR sorting has been shown to be regulated by a subset of ESCRT proteins comprising Hrs and Tsg101, which are subunits of ESCRT-0 and ESCRT-I respectively, and the ESCRT accessory protein Alix [106]. The requirements for Alix and Hrs were not surprising, since both of these ESCRT-associated proteins had already been linked to non-conventional endosomal sorting: Alix by mediating ubiquitin-independent MVB sorting of P2Y_1_ purinergic receptors; and Hrs through recognition of hydrophobic amino acid clusters regulating degradation of cytokine receptors [108,109]. Although its role remains unclear, Tsg101 reportedly controls endosome-to-cytosol release of enveloped viral RNA through an interaction with Alix, implicating a potential role in ILV back-fusion [110]. 

A number of EGFR signaling pathways have been linked to NFκB activation downstream of ligand stimulation or constitutive EGFR activation in cancer cells, primarily by promoting degradation of IκB proteins that sequester inactive NFκB proteins in the cytoplasm [79,111]. However, our recent studies provide the first evidence that stress-induced EGFR signaling contributes to the innate immune response to adenovirus by interfering with negative feedback control of NFκB activity in the nucleus (Figure 2). We found that viral cell entry induced EGFR phosphorylation at the p38-MAPK/MK2 substrate Ser1046/1047, which was previously linked to clathrin-mediated uptake of stress-exposed EGFRs [73,112]. We also found that adenovirus-induced EGFR trafficking occurred in the absence of EGFR ligand, as well as EGFR ubiquitination and intrinsic tyrosine kinase activity that are both required in the ligand-regulated trafficking pathway [113,114,115]. Nevertheless, EGFRs were subsequently activated, and NFκB-p65 was phosphorylated at a Thr254-Pro motif, which is a known substrate for Pin1, by a mechanism that was attributable to intrinsic EGFR kinase activity [73]. Although the EGFR-stimulated pathway regulating phosphorylation of the Thr254-Pro motif in NFκB-p65 is not currently known, the Gab1 signaling adaptor is a strong candidate. In addition to requiring trafficking to endosomes for full activation, Gab1 is known to sustain EGFR/ERK-MAPK signaling by facilitating activation of the tyrosine phosphatase Shp2 [85,86,116,117,118]. There is increasing evidence that many viruses exploit EGFR function to facilitate their replication, and antagonize host antiviral responses [119]. Until recently, it was generally assumed that viruses co-opted mechanisms induced by ligand-receptor interactions. However, it is well-established that viral infections can impose cellular stress and minimize or co-opt adaptive host responses [120]. Recognition that adenovirus contributed to NFκB signaling by activating a novel stress-induced EGFR pathway is significant, because unique host proteins regulating this pathway represent novel drug targets for therapeutic development.

## 6. Innate Immunity and Early Adenoviral-Induced Gene Products

Adenoviral proteins encoded by early region genes elicit a second wave of NFκB activity 3 to 4 h post-infection. Respiratory epithelial cells are normally unresponsive to bacterially-derived LPS (lipopolysaccharides), which is present as a contaminant on airborne particles and activates NFκB following its binding to TLR4 (Toll-like receptor 4) [121]. However, multiple lines of evidence indicate that the adenoviral E1A (early region 1A) protein sensitizes infected respiratory epithelial cells to LPS/TLR4/NFκB signaling. First, some investigators have suggested a role for persistent E1A expression in COPD [122]. Second, primary human bronchial epithelial cells that stably expressed the adenovirus E1A protein displayed enhanced pro-inflammatory NFκB signaling, compared to untransfected control cells [123]. Finally, enhanced susceptibility to LPS and other microbial inducers of inflammation have been implicated in the acute inflammatory responses to adenovirus infections and toxicity of adenovirus vectors used in gene therapy [124,125]. A second early viral gene product with known involvement in NFκB signaling is the 19K protein encoded by the E3 region (E3-19K). E3-19K is a membrane protein that binds and retains MHC class I molecules in the ER, suppressing anti-adenovirus activities of T cells [1,126,127,128]. This interaction subsequently triggers ER protein overload, leading to calcium release, production of reactive oxygen intermediates, and NFκB activation [129]. In addition to early viral gene products, NFκB activity is also regulated by TNF-α released from adenovirus-stimulated alveolar macrophages, and recognized by TNFR1 (Tumor necrosis factor receptor 1) located on the apical surface of respiratory epithelial cells (Figure 1d) [93,130]. In addition to the TNFR1 adaptor molecule TRADD, which assembles a multi-adaptor complex inducing IκB degradation, we have shown that TNF-α controls nuclear NFκB stability through the stress-activated EGFR signaling pathway described in adenovirus-infected cells [73,131]. In contrast to virally-induced events that have a positive role in NFκB signaling, the E3-14.7 adenoviral protein inhibits NFκB gene transcription occurring downstream of both LPS and TNF-α, through a direct interaction with the NFκB p50 subunit that inhibits DNA binding [132]. The NEMO/IKKγ regulatory subunit of the IKK complex has also been reported to be an E3-14.7 binding partner [133]. However, this physiological relevance of this interaction remains unknown, since it had no effect on NEMO/IKKγ function [133]. Adenovirus also encodes two viral-associated RNAs (VA-I and VA-II), that are synthesized during the early phase through the late phase of infection [134]. Recent studies have revealed that these non-coding RNAs induce the production of type I interferons, but not inflammatory cytokines [135].

## 7. Innate Immunity and the E3-RIDα Protein

**Figure 3:**
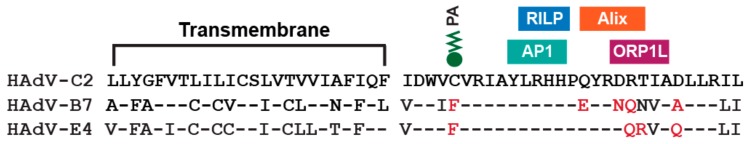
Sequence alignment of published RID-α transmembrane and cytosolic tail sequences from adenovirus serotypes HAdV-C2, HAdV-B7, and HAdV-D4. HAdV-C2 RIDα is known to interact with multiple cellular proteins described in the text, and is also reversibly modified with palmitic acid (PA) at Cys67. Divergent amino acids affecting the Cys67 palmitoylation site, or introducing negative/positive to uncharged amino acid substitutions, are highlighted in red.

We have characterized three independent roles for the E3 gene product E3-RIDα encoded by group C adenoviruses (HAdV-C2 and HAdV-C5) in modulating NFκB activity in cultured respiratory epithelial cells (Figure 1d). E3-RIDα is a type II transmembrane protein localized to early endosomes, where it regulates sorting of both membrane protein and lipid cargoes [114,136,137,138]. Our studies have identified several small modules composed of 2–6 residues located in the cytosolic E3-RIDα C-terminal region, regulating functional interactions with the following host proteins or protein complexes (Figure 3): (i) The clathrin adaptor AP1, which assists in the temporal regulation of the viral protein by facilitating its localization to endosomes [139]; (ii) the ESCRT accessory protein Alix involved in EGFR trafficking induced by cell stress, including adenoviral infection [73]; (iii) the Rab7 effector Rab-interacting lysosomal protein (RILP), which controls late endosome transport and positioning, and late endosome-lysosome fusion [140,141]; and (iv) a second Rab7 effector called ORP1L (Oxysterol-Binding Protein-Related Protein 1L), which supports many RILP functions, and also contributes to lipid trafficking at membrane contacts between late endosomes and ER [138,142,143]. We have shown that the C-terminal tail of the viral protein is intrinsically disordered, suggesting E3-RIDα function is regulated by disorder-to-order transitions induced by binding to its various protein partners [144]. The dynamic nature of these interactions is also supported by the finding that E3-RIDα function relies on the reversible attachment of palmitic acid to Cys67 in its cytosolic tail (Figure 3) [145]. Although the role of this modification is not currently known, it could elicit conformations that are favorable for binding host cell proteins, or regulate E3-RIDα targeting to endosomal lipid rafts [146].

The first mechanism involves the ability of E3-RIDα to attenuate the stress-induced EGFR signaling pathway that antagonizes negative feedback control of nuclear NFκB activity in respiratory epithelial cells (Figure 2) [73]. Our published results indicated that EGFRs were diverted away from the Tsg101-dependent trafficking step in the stress-induced MVB sorting pathway, following the onset of early viral gene expression [73]. Phosphorylation at the Thr254-Pro Pin1 substrate in the p65 subunit of NFκB was also inhibited, with a corresponding reduction in NFκB-p65 nuclear stability and NFκB gene transcription [73]. In addition, E3-RIDα expression was associated with down-regulated EGFR/NFκB signaling induced by TNF-α in the absence of other viral proteins, supporting a role for E3-RIDα in infected cells [73]. These findings also suggested that ILV back-fusion required for EGFR signaling from MVB limiting membranes was somehow blocked. Our finding that E3-RIDα formed a physical complex with the ESCRT accessory protein Alix support a working model that the viral protein could interfere with a back-fusion process regulated by an interaction between Alix and Tsg101, as has been suggested for endosome-to-cytosol release of enveloped viral RNA [110]. It was already known that Pin1 modulates NFκB signaling following its release from IκBα, which exposes the Thr254-Pro motif in the p65 subunit, in cytokine-treated cells [86]. Similarly, EGFR-dependent phosphorylation of the NFκB-p65 Thr254-Pro motif in infected cells probably requires IκBα degradation downstream of PI3K/Akt signaling, which is probably induced by PI3K/Akt signaling downstream of capsid binding to αV integrins [67]. It seems unlikely that E3-RIDα-mediated reduction of stress-induced EGFR signaling would have much of an effect on this first wave of NFκB activity downstream of viral cell entry, if cells are infected at relatively low multiplicity of infection (MOI). However, Pin1-deficient mice and cells are refractory to NFκB activation by cytokine signals, suggesting that E3-RIDα could have a significant impact on NFκB activity induced by exposure to TNF-α released by adenovirus-activated macrophages [86]. On the other hand, failure to down-regulate EGFR stress signaling could lead to constitutive NFκB activity, by blocking its nuclear export in the absence of external stimuli.

A second mechanism for regulating NFκB signaling involves the interaction between E3-RIDα and the Rab7 effector molecule RILP [147]. In some of our earlier studies, we noted that infection with a mutant virus lacking E3-RIDα expression had a gross morphological effect on the endolysosomal network [145]. The aberrant phenotype was characterized by cholesterol accumulation in dysmorphic perinuclear compartments enriched for early and late endosomal markers [145]. By comparison, endolysosomal compartments were largely devoid of free cholesterol, and were similarly distributed in the cytoplasm, in mock-treated and HAdV-C2-infected cells [145]. We had also previously established that E3-RIDα expression was required to divert internalized EGFRs from a recycling to a lysosomal degradative pathway in infected cells [113,114]. However, adenovirus-induced EGFR degradation was not impaired by reduced function of the small GTPase Rab7, which is critically important for late endosome/lysosome function [73,148]. Interestingly, we had isolated RILP, which couples Rab7-positive vesicles to the HOPS (homotypic fusion and vacuole protein sorting) tethering complex required for late endosome-lysosome fusion, in a yeast two-hybrid screen for E3-RIDα binding partners [147]. To determine whether the interaction between E3-RIDα and RILP activated an alternative degradative pathway, cells were transfected with a plasmid encoding a dominant-inhibitory RILP fragment, which blocks the interaction between RILP and HOPS without affecting RILP binding to E3-RIDα [140,147]. We found that this fragment led to a significant reduction in EGFR degradation associated with E3-RIDα expression by infectious virus [147]. In addition, the wild-type E3-RIDα protein, but not a mutant protein defective for RILP binding, was sufficient to reconstitute lysosomal degradation of LDL (low density lipoprotein) following its receptor-mediated uptake in Rab7-depleted cells [147]. E3-RIDα expression also reconstituted ligand-induced EGFR degradation in cells with reduced Rab7 function [73]. Altogether, these results support a working model that the interaction between E3-RIDα and RILP facilitates a lysosome sorting mechanism normally regulated by Rab7, which was disabled by adenovirus infection. Although the mechanism of Rab7 inactivation is not currently known, it probably occurs downstream of one of the myriad pathways that are activated by viral cell entry. For instance, Rab7 is emerging as a target for signaling pathways that disrupt its interaction with RILP through Rab7 phosphorylation [149].

Impaired Rab7 function would be beneficial for viral replication, by preventing adenovirus clearance in lysosomes. However, the reported ability of infected cells to evade premature death from TNF-α-induced apoptosis by down-regulating TNFR1 requires a functional endolysosomal system [150]. Adenovirus-induced TNFR1 down-regulation is known to be regulated by a functional interaction between E3-RIDα and a second E3 protein called E3-14.5 (or RIDβ) [150,151,152]. We hypothesize that TNFR1 down-regulation occurs in a two-step process. The first step involves clathrin-mediated TNFR1 internalization, possibly through an interaction between E3-14.5 and AP2 clathrin adaptors [151]. Our data suggest that E3-RIDα directs the second step in this virally-induced pathway, by promoting RILP-dependent late endosome-lysosome fusion in the absence of functional Rab7. In contrast to adenovirus-induced TNFR1 trafficking, E3-RIDα is the only viral protein required for EGFR down-regulation in infected cells, because clathrin-mediated EGFR internalization is regulated by a stress-induced physiological pathway [73,153]. Similarly, E3-RIDα expression supported EGFR degradation in Rab7-depleted cells in the absence of other viral proteins, following ligand-stimulated EGFR internalization from clathrin-coated pits [73].

**Figure 4:**
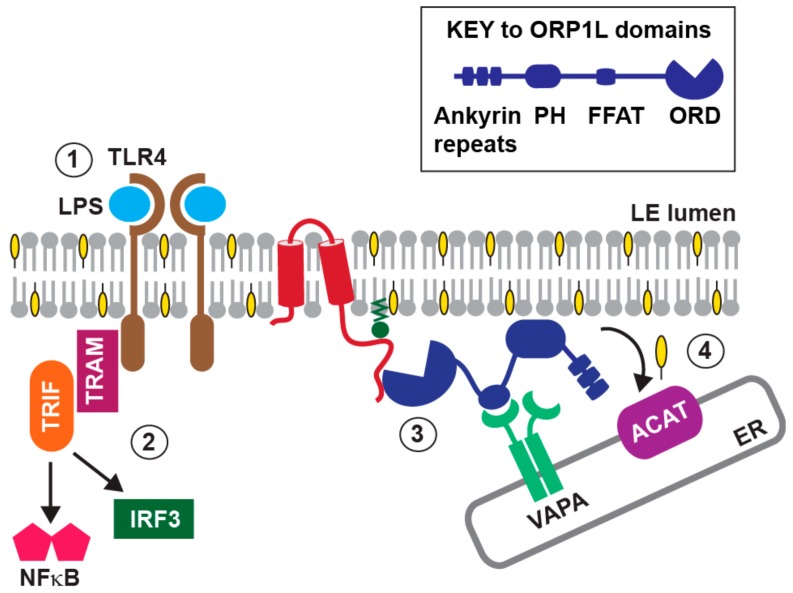
Hypothetical model for adenoviral regulation of cholesterol-dependent TLR4 signaling. It is already established that LPS-activated TLR4 traffics to endosomes, where it is associated with cholesterol (yellow)-enriched lipid rafts (1); and that endosomal TLR4 regulates NFκB and IRF3 signaling by engaging TRAM/TRIF adaptors (2). Our studies support a working model that E3-RIDα promotes formation of ORP1L-VAP membrane contacts (3), which regulate cholesterol-dependent TLR4 signaling through cholesterol transport to ER for esterification by Acyl-coenzyme A: cholesterol acyltransferase (ACAT) (4).

The third mechanism involves the interaction between E3-RIDα and ORP1L, a lipid binding protein belonging to the family of oxysterol-binding protein related-proteins (ORPs) [154,155,156,157,158]. In addition to its evolutionarily conserved lipid binding ORD (OSBP-related domain), ORP1L has amino-terminal ankyrin repeats mediating its interaction with GTP-Rab7, a pleckstrin homology (PH) domain partially responsible for targeting to late endosomes, and an FFAT [two phenylalanines (FF) in an Acidic Tract] motif (Figure 4) [159,160]. The ORP1L-ORD has been shown to exist in two conformations in partnership with GTP-Rab7-GTP: A membrane-bound state when late endosomal cholesterol levels are high; and a cytosolic, membrane-free conformation enabling formation of late-endosome-ER membrane contact sites via an interaction between ORP1L-FFAT and VAP (VAMP [vesicle-associated membrane protein]-associated ER protein) located in the ER [160]. In contrast, we have shown that E3-RIDα stabilized the interaction between ORP1L and the VAP isoform VAPA under endosomal cholesterol-loading conditions that normally inhibit ORP1L/VAPA complex formation [138]. In addition, these E3-RIDα-induced membrane contact sites supported the non-vesicular flow of endosomal cholesterol down a concentration gradient to the ER, where it was rapidly converted to cholesteryl esters for storage in cytoplasmic lipid droplets by the enzyme Acyl-coenzyme A: cholesterol acyltransferase (ACAT) (Figure 4) [138]. We discovered the RIDα-dependent cholesterol transport pathway by expressing the viral protein in human fibroblasts from a patient with NPC (Niemann-Pick type C disease) bearing mutations in the NPC1 protein [145]. NPC1 is known to regulate the predominant late endosome-ER cholesterol transport pathway, and the main phenotype in cells from NPC patients is the accumulation of cholesterol and other lipids in the endosomal/lysosomal compartment [161]. We found that the interaction between E3-RIDα and ORPIL corrected this aberrant cellular phenotype in the absence of other viral proteins, by a mechanism requiring the Cys63 palmitoylation site in the E3-RIDα cytosolic tail [145]. It had been reported previously that NPC1 deficiency was associated with constitutive inflammatory signaling through TLR4 [162]. Studies had revealed that TLR4 is localized at both the cell surface and in endosomes, and that endosomal TLR4 recruits specific signaling adaptors (TRAM and TRIF) linking it to IRF3 and NFκB transcription factors [163,164]. It was also known that TLR4 associates with lipid rafts, and that its activity was modulated by levels of cellular cholesterol [165,166]. These properties of TLR4 may underlie its constitutive activity in NPC1-deficient cells, because NPC1-mediated cholesterol transport is required to deplete raft components from late endocytic membranes [162,167]. Since infected cells developed an NPC-like cholesterol storage phenotype following infection with an E3-RIDα-null virus, we hypothesized that the interaction between E3-RIDα and ORP1L reconstituted homeostatic control of TLR4 signaling in endosomes [145]. Consistent with this hypothesis, LPS induced an acute transitory NFκB response in respiratory epithelial cells infected with wild-type virus [143,145]. In contrast, mutant viruses lacking E3-RIDα, or encoding a palmitoylation-defective E3-RIDα protein, were both associated with sustained NFκB activity [143,145]. Prior to our studies, ORP1L was known to be involved in cholesterol sensing, but had not been linked to cholesterol transport [160]. However, recent studies have identified a physiological pathway for cholesterol transport regulated by the interaction between ORP1L and VAPA [142]. Our recent results suggest this physiological pathway may also have a key role in homeostatic TLR4/NFκB signaling.

## 8. Conclusions and Future Perspectives

To perform its important role in epithelial cells, NFκB activity must be tightly regulated to ensure that: (1) its cytoprotective functions are preserved; (2) pro-inflammatory responses are rapidly induced by pathogen challenge; and (3) the initial pro-inflammatory response is down-regulated to prevent excessive tissue damage and injury [65]. Many human pathogens have evolved strategies to modulate NFκB activity by targeting mechanisms regulating IκBα degradation downstream of inducible IKKs or IKK-like kinases [168]. Our recent studies indicate that the E3-RIDα protein also targets the NFκB pathway, but by mechanisms that had not been previously described [73,143].

Although E3-RIDα was originally identified for its ability to down-regulate EGFR, this activity had not definitively been associated with a biological function [114]. We have now shown that E3-RIDα antagonizes stress-induced EGFR signaling associated with enhanced NFκB stability and gene transcription. Since this stress-induced EGFR pathway occurred downstream of adenoviral cell entry, its impact on innate immunity could be related to MOI. On the one hand, low MOIs will support viral replication during a primary infection [169]. Under these conditions, the stress-induced EGFR pathway may primarily regulate NFκB activity that is cytoprotective. On the other hand, one viral particle can replicate up to 10^6^ new progeny leading to high MOI secondary infections in surrounding tissue [170]. Although these secondary infections induce cell death before the virus can replicate, they may also cause an exaggerated EGFR stress response contributing to adenoviral pathogenesis, if left unchecked by E3-RIDα expression [171]. Our studies suggest that E3-RIDα attenuates stress-induced EGFR signaling by orchestrating a novel two-step process. The first step appears to block ESCRT machinery regulating EGFR signaling from MVB limiting membranes. The second step then facilitates lysosomal degradation by a mechanism that may compensate for loss-of-Rab7 function induced by viral cell entry.

NFκB activity is deregulated in various inflammatory diseases, making the NFκB signaling pathway an attractive target for anti-inflammatory therapies. Several categories of inhibitors have been developed, which block different steps in the canonical NFκB activation cascade [172]. The finding that stress-activated EGFRs regulate NFκB activity via a mechanism involving a host cell Pin1 substrate could provide a foundation for more selective therapies with Pin1 inhibitors, which are currently being developed for cancer therapy [172]. Further examination of Pin1 substrates, either in viral proteins with critical roles in replication, or host cell pathways contributing to innate immunity, represents an important topic for future investigation.

In addition to its role in down-regulating a stress-induced EGFR/NFκB pathway, we have found that E3-RIDα modulates NFκB activity downstream of the LPS receptor TLR4, through formation of membrane contact sites controlling endosomal cholesterol levels. Virus-induced membrane contact sites are emerging as key players in the formation of specialized membranous replication organelles with unique lipid composition, which facilitate robust replication of positive-strand RNA viruses [173]. However, to our knowledge, our studies are the first to link membrane contacts to innate immune signaling associated with a pathogen-induced pathway. We have also shown that E3-RIDα-stabilized ORP1L/VAP complexes appear to keep ER cholesterol concentrations low enough to alleviate negative feedback inhibition of SREBP1 (sterol regulatory element binding protein 1) transcription factor activation [143]. Since SREBP-1 regulates production of fatty acid metabolites that antagonize NFκB-dependent transcription, this may provide another means of fine-tuning NFκB responses to adenovirus infection [174].

E3-RIDα activities could also be important factors in adenovirus disease associated with specific adenovirus strains such as HAdV-B7 and HAdV-E4, which account for several recent highly publicized outbreaks [21,175]. In contrast to *E1A* and early region 1B (*E1B*) genes that are conserved among different adenovirus serotypes, *E3* genes vary markedly and may even be absent, supporting their potential roles in adenovirus pathogenesis [27]. For instance, adenovirus E3-19K proteins encoded by different serotypes display allele-specific interactions with class I MHC molecules, implying a link between host genetic factors and susceptibility to adenovirus disease [176,177]. E3 RIDα proteins from different serotypes exhibit significant sequence divergence in the C-terminal region encoded by HAdV-C2, which mediates interactions with host proteins involved in membrane trafficking (Figure 3). For instance, HAdV-B7 and HAdV-E4 have negative/positive to uncharged amino acid substitutions in the putative Alix binding site, which could potentially impact the strength and duration of EGFR signaling induced by stress of viral infection or exposure to TNF-α. They also lack the Cys67 palmitoylation site regulating endosome-ER contact site formation, suggesting they may be associated with unregulated LPS/TLR4/NFκB signaling.

Although HAdV-C serotypes are generally associated with undiagnosed or mild symptoms, they are also clinically relevant in causing severe manifestations in immunocompromised patients, particularly in allogeneic hematopoietic stem cell transplant recipients [178,179,180]. Whole genome sequences were recently reported for 51 circulating clinical strains derived by recombination between different HAdV-C subtypes [181]. Those studies revealed that the majority of recombination events involved E1 and E4, implicating these early transcription regions in HAdV-C virulence [181]. In contrast the entire E3 region was highly conserved, suggesting there is strong selective pressure to maintain E3 sequences [181]. It is well established that HAdV-C DNA can persist in a latent state in lymphoid cells for many years, and a role for E3-RIDα and E3-RIDβ in preventing apoptotic cell death through TNFR1 and related death domain-containing receptors has been postulated [182,183]. These findings suggest that E3-RIDα encoded by HAdV-C may support reactivation of latent virus, and subsequent systemic infections, in immunosuppressed transplant patients [184].

E3-RIDα may also have important roles in replication-selective oncolytic adenovirus vectors. In addition to a small deletion in E1A that abolishes binding to the retinoblastoma protein, the oncolytic adenovirus vector *dl*922-947 has a second deletion eliminating expression of E3 proteins RIDα and RIDβ [185]. It was predicted that this E3 deletion might make this virus more sensitive to the antiviral effects of TNF-α. However, recent studies have shown that that *dl*922-947 induces a novel form of TNF-α-independent programmed cell death, which differs from classical apoptosis and necroptosis pathways [186]. Our studies suggest an alternative mechanism involving the interaction between HAdV-C-encoded RIDα and ORP1L illustrated in Figure 4. Loss of homeostatic RIDα signaling through ORP1L in cells infected with *dl*922-947 could lead to cholesterol-induced cytotoxicity, which is known to trigger a complex network of cell death pathways [187]. In addition, cholesterol accumulation in macrophages and other immune cells may provoke inflammatory responses that could have oncolytic effects in distant non-virally infected tumors [188]. In addition to co-option of cytokine-regulated inflammatory pathways that has been a primary focus of E3 biology for nearly three decades the role of cholesterol in innate immune responses to adenovirus vectors is an important topic for future investigation. 

To summarize, E3-RIDα is a remarkably versatile adenoviral membrane protein localized to endosomes, which is capable of targeting the NFκB signaling pathway by multiple mechanisms. Different pathogens tend to converge on similar pathways, usually by deploying distinct strategies. Studying how other pathogens potentially subvert the novel host cell pathways targeted by E3-RIDα will provide fundamental new information regarding basic molecular cell biology, and is also central to understanding infectious disease.
